# Defeater Goes External

**DOI:** 10.1007/s11406-016-9803-y

**Published:** 2017-02-22

**Authors:** Mikael Janvid

**Affiliations:** Department of Philosophy, Stockholm University, 106 91 Stockholm, Sweden

**Keywords:** Defeater, Externalism, Warrant, Reliable indicator, Misleading defeater

## Abstract

This paper proposes a new externalist account of defeaters, in terms of reliable indicators, as an integral part of a unified externalist account of warrant and defeat. It is argued that posing externalist conditions on warrant, but internalist conditions on defeat lead to undesirable tensions. The proposal is contrasted to some rival accounts and then tested on some widely discussed cases, like the airport case. Misleading defeaters, where Laurence BonJour’s reliable clairvoyants serve as examples, also receive treatment, partly because they illustrate how internalist constraints are inserted into the set up of the problem and therefore unduly constrain the domain of satisfactory solutions. Lastly, the proposal is defended against some objections. Firstly, that by posing externalist conditions on defeat, the account becomes too open. Secondly, that an externalist account fails to take into account the epistemic assessments of our fellows in the epistemic practice of forming beliefs and making epistemic claims, which can be based on accessible warrant only.

## Introduction

These days most epistemologists pay tribute to the defeasible nature of warrant.^1^ But, what is a defeater more precisely and when does defeat occur? As is often the case with views that have become platitudes, these important features are not so clearly understood as one would wish. Moreover, an epistemological position that only investigates warrant, the positive epistemic aspect, seems truncated. I am convinced that investigating defeat, “the opposite side of warrant”, deserves as much attention as warrant has received within epistemology. The positive and negative aspects unite for all those positions that include a “no defeater”-condition on warrant.

Most existing accounts pose internalist conditions on defeat, even those given by otherwise externalist minded epistemologists. Unsurprisingly, externalist positions with some internalist condition included get more complicated than uniform models and, more seriously, give rise to internal tensions, as we shall see.^2^ A working hypothesis of this paper is that in assessing the epistemic status of a subject’s belief that p, the same conditions should be used in what counts as favoring the subject to be in a positive epistemic state as well as what counts against the subject being so positioned.

A variety of different approaches to defeat are already in play. Due to their diverging starting points, a detailed survey and comparison between every one of them to the upcoming proposal would not get us beyond the starting points. Even if the presuppositions are important, it is also imperative to consider their development towards a full proposal. The upcoming proposal will thus be compared only to a selection of those, which fruitfully explore contrasts and objections raised against it. Furthermore, in this paper I often employ the general term “positive epistemic status”. My proposal, however, concerns the epistemic constituent of knowledge, here called warrant, rather than the state of knowledge itself. I thus do not aim to cover Gettier cases with my proposal, i.e. cases where the subject has a true warranted belief that p, but nevertheless does not know that p.

The aim of this paper is to capture the epistemic features, which determine whether a subject enjoys such a positive epistemic status vis-à-vis some belief or if she has that status defeated. My proposal does *neither* provide guidelines for an epistemically rational subject, nor what such agents should be prohibited or permitted to believe. Of course, within certain epistemological theories these aims coincide. Here externalism (but not only that position) separates them with respect to positive epistemic status and I propose to do the same with respect to the epistemic properties that defeat those states. The arguments counting in favor of doing so in the former case, do so in the latter as well, or so I shall argue.

If one so wishes, one could see the upcoming investigation as providing prior groundwork for a model of epistemic rationality, to which internalist constraints and guidelines could be added. More precisely, when an agent is confronted with an incoherent system of beliefs, the upcoming integrated account will provide uniform conditions on what beliefs in the set more precisely are warranted or defeated and then the agent can, to the best of her abilities, in the next instance try to revise them accordingly. Since agents most often are not in epistemically ideal conditions, they will have to do with what is available to them at the time, just like the case where only positive epistemic features are considered. The upcoming account thus aims to specify what it would be like to be a fully informed subject in these matters, what factors subjects would need to have access to in order to be ideally situated epistemically.

In the absence of such an investigation, mere incoherence within the system of beliefs as such does not tell us which belief, if any or all, is defeated. Not providing conditions on defeat invites radically holistic Quinean scenarios where the subject is free to treat any belief as defeated (or none for that matter). By reducing epistemic status to solely consisting in managing rational internal coherence, we ignore basic and necessary aspects of the epistemic status of a subject in both the positive case of warrant as well as the negative case of defeat, one of them being the *strength* of warrant of the involved beliefs.

If I were to choose a reader of this paper, it would precisely be an externalist with internalist leanings regarding defeat to convince her that my upcoming proposal can incorporate some of these traits, but, nonetheless, support them in an externalist fashion.^3^

The structure of the paper is as follows: the next section introduces an important distinction between two different kinds of defeaters as well as the notion of a misleading defeater as illustration. Both serve as preliminaries for my upcoming proposal in the third section. In the fourth and final section of the paper two objections are raised and answered, prompting further elaboration of the proposal.

## Varieties of Defeat

We begin the investigation of defeaters by drawing an important distinction between two different kinds: an *overriding* defeater to an alleged positive epistemic status for a belief that p provides warrant for non-p, while an *undermining* defeater to p defeats the warrant that the belief that p is based on. In the latter case, no warrant has thereby been provided for non-p.^4^ More precisely, where warrant is a relation r, having at least two *relata* (i) the ground, or evidence, g, and (ii) the belief that p, then an undermining defeater can either defeat g, or defeat that g stands in r to p.

Sometimes defeating g may override p, as in the case of an inductive inference concerning the color of swans: The observation of a non-white swan overrides the generalization that all swans are white. In those cases we have instead come across an overriding defeater. A relevant example of defeating g, and thereby undermining the warrant for p, would be where an alleged sighting of a white swan indeed was of a white bird but not a swan. If we instead phrase g in that case as “There is a white bird over there”, then g would be true, but not stand in relation r to p. An example of defeating r would be to base your belief that all birds of a certain species have a uniform color on the observation of a sample that later turns out to be too small due to the discovery of another population or that all birds in the observed sample turn out to belong to a subspecies. It could also be the case that this species of birds are later discovered to change colors during mating season, as male mallards (partly) do. Then g remains true, but no longer provides sufficient warrant for p. Since warrant is a state admitting degrees, the relation r thus allows for degrees, but has a threshold that is no longer reached in the latter example.

I now turn to the internalist constraint of accessibility on defeat. Instructive in this regard is the problem of misleading defeaters. Say that a subject is in the epistemic state of knowing that p, but is faced with a challenge to p.^5^ Can the challenge, thereby, solely on account of the subject knowing that p, without further consideration, be dismissed as misleading? Such a dismissal seems dogmatic and, yet the subject actually knows that p!

Here one might wonder how a misleading defeater can both mislead *and* defeat. It would seem that it could only do one of these things: if the challenge misleads, then it does not defeat. But if the challenge defeats, then it does not mislead. The reason a misleading defeater seemingly can do both is due to the internalist stage setting of the problem where the misleading nature of the defeater is not accessed by the relevant subject and *therefore* defeats her alleged positive epistemic status. Misleading defeaters thus follow a formula where relevant evidence revealing the misleading character of the defeater is inaccessible to the subject at one point of assessment. On the basis of the accessible evidence, the targeted epistemic status is then defeated. Notice, however, that even from an internalist perspective, there is no point in time in which a challenge synchronically can be *both* misleading and constitute a defeater for a particular subject. Depending on the warrant brought into consideration, the verdict is always either or. In order to investigate this phenomenon more carefully, but without claiming to address the puzzle in its entirety, we need to make the internalist tacit assumptions explicit and only then consider some examples.

While I shall below in section 4 acknowledge the prima facie internalist character of the epistemic practice of justifying and challenging epistemic claims and thus that a subject faced with a misleading defeater would prima facie face defeat of her belief, the *ultima facie* character of a misleading defeater is precisely that of being misleading rather than a (genuine) defeater. In a relevant sense specified below, on my proposal what decides the epistemic status of the challenged claim in question is its misleading nature, irrespective of whether its misleading character is accessible to the subject or not. Due to the aforementioned tendency to accept internalist constraints on defeaters, even most externalists do not reach this verdict, which, will be argued below, is the correct one.^6^

Before we can reach this verdict, however, a number of issues need to be dealt with. We need, for instance, to specify precisely what kind of defeater is in play in each case under consideration. If knowledge is present, then the warrant, on which the belief that p is based, is, as we shall see, on my upcoming externalist account robust against undermining defeaters in a way in which the belief that p itself is not against overriding defeaters. Whereas warrant in virtue of being sufficient for knowledge possesses objective truth conduciveness apart from the truth of p, the truth of p itself is compatible with the existence of warrant for non-p. Contrary testimony is but one example. Undermining challenges may of course be present, but does not defeat the knowledge that p – otherwise knowledge would not be at hand in the first place according to my proposal. A subject would not have come to know that p unless the warrant the subject based her belief that p on were objectively truth conducive. We shall return to this point in section 4 below. In the case of overriding challenges by contrast, they can sometimes defeat knowledge even though the warrant for the belief that p is not likewise defeated. From a fallibilist point of view, this may happen when the warrant is strong enough to pass the threshold of knowledge, but is not conclusively warranted. An interval thus exists in between for overriding defeaters to place themselves. (A point we shall return to as well in section 4.)

As illustrations, it is underappreciated that at least two of the four clairvoyants, famously introduced by BonJour,^7^ are examples of misleading defeaters; more precisely one of each kind of defeater. The first case figures Samantha who without having any reasons for or against her conviction that she is endowed with clairvoyant powers trusts in them concerning the whereabouts of the American president despite being faced with overriding challenges locating the president elsewhere. In the third case, Maud faces all the suspicions, supplied by friends and relatives, we nurture against the general reliability of clairvoyance, thus an undermining challenge (more precisely to r in the terminology introduced above). Without any reasons in favor of her possessing clairvoyant powers, she forms a belief concerning the whereabouts of the American president. These suspicions notwithstanding, both Samantha and Maud are, in fact, *reliable* clairvoyants and their beliefs are both true. Both of them are thus faced with misleading defeaters. If Maud were not confronted with these false suspicions, she would be like Norman, the fourth case BonJour introduces. Norman lacks *any* reasons for or against being equipped with clairvoyant powers, but he, nonetheless, forms a true belief based on his likewise reliable belief-generating mechanism.

Bonjour regards Samantha, Casper, Maud and Norman as providing counterexamples to reliability providing a sufficient condition on warrant. His discussion of the case of Norman reveals that Bonjour poses the additional requirement on warrant, not only that challenges are lacking against their clairvoyant powers and visions, but also positive warrant in favor of these powers being reliable, as they actually are in all these cases. This strong internalist requirement pinpoints a dividing line between BonJour and externalism in that the latter rejects this requirement as constituting a “hyper-intellectualization” of epistemic states,^8^ which, moreover, is impossible to meet in a considerable amount of cases due to epistemic circularity. From an externalist perspective, Norman is in the same predicament regarding his clairvoyant powers as many people are regarding their perceptual abilities. We cannot deny that Norman is warranted in his clairvoyant beliefs unless we are also willing to deny that these people are in positive epistemic states regarding their perceptual beliefs without having collected evidence, at least as track records, in favor of the reliability of their perceptual mechanisms.^9^ Before the aforementioned verdict on the clairvoyants as warranted in their beliefs (with a *proviso* in the case of Samantha elaborated on in section 4), despite lacking positive warrant for their reliability, and the alleged defeaters as misleading rather than genuine, can be further substantiated for, I first need to present the heralded externalist condition on defeat.

Once more, it seems highly plausible that the features that determine the epistemic status of a belief are the same, irrespective of whether it is done positively, in the case of warrant, or negatively, in the case of defeat. Let me specify two important aspects. First, if we consider the truth conduciveness of a ground as decisive for determining positive epistemic status, irrespective of whether the subject has access to that ground, then why should we not do so regarding factors counting *against* the subject gaining such status, i.e. defeat? Here the burden of proof seems to lie on those who deny the parallel. Second, if we object to hyper-intellectualized internalist requirements on warrant, why should we not raise the same objection when it comes to such requirements on defeat? Perhaps hyper-intellectualism regarding defeat would be legitimate if accounting for defeat solely consisted in maintaining intrapersonal rational coherence, but, to repeat, we should reject treating defeat in that narrow and misleading way.

The internalist constraints many externalists pose on defeat on the negative side thus stand in tension to the externalist ones on the positive side. To come up with a unified externalist account of both these epistemic properties is therefore a *desideratum* for externalism. Or, for that matter, if one would like to pose a “no defeater”-condition on warrant itself, as part of a totality constraint on warrant, as I wish to do, then that clause should be grounded in externalist fashion as well. Such an externalist alternative is proposed in the next section.

## The Proposal

I begin with the no defeater-condition. The key idea behind an externalist grounding of a no defeater condition is that aside from the condition of positive warrant for a belief, the addition of a no defeater condition, when fulfilled, *thereby* provides a higher degree of truth conduciveness, and thus of total warrant for that belief, than if a no defeater condition was not included.^10^

An *undermining* defeater to p consists of truth conducive indicators of g or r *not* holding; again without any condition requiring the subject to have access to that defeater. They defeat the warrant for p iff they provide stronger indicators of g or r not holding than the positive warrant for g or r provide.

An *overriding* defeater to p is, next, explicated in externalist fashion as the existence of truth conducive, reliable *indicators* of non-p, irrespective of whether the subject has access to that defeater or not. These indicators defeat the positive epistemic status of the belief that p iff these reliable indicators of non-p are at least as strong or stronger than the reliable indicators of p that the positive warrant consist of. Warrant needs to reach a certain threshold (I shall regrettably leave aside where precisely the threshold is located as well as whether it is variant or not), but even where positive warrant succeeds in doing so, overriding defeaters may still defeat the positive epistemic status of the belief that p.

When no (sufficiently strong) defeaters of any kind are present, then the no defeater condition on warrant is fulfilled. A subject is thus warranted in her belief that p iff there are sufficiently strong reliable indicators for g, r and (thereby) p holding *and* they are stronger than any against them holding.

Defeaters are thus tied in externalist fashion to the presence of reliable indicators of their respective kind of defeat, irrespective of any internalist requirement on access to them. In the aforementioned examples concerning bird sightings, the presence of a black swan is a reliable indicator of the negation of the belief that all swans are white. In case of undermining defeaters, when you base that belief on the observation of a white bird that turns out not to be a swan, then your belief that g is overridingly defeated, which in turn undermines your warrant for your belief that all swans are white. Next, the belief that all birds of a certain species have a uniform color based on a sample of observations that, due to aforementioned later discovered external circumstances, turns out to be too small is no longer a reliable indicator for your belief and thus that r does not hold between g and p (even though g in this case holds since the sample indeed consists of birds with that same uniform color). Thus in the case of undermining g, warrant exists for non-g, but in the case of r, the defeater consists in lack of r holding rather than necessarily warrant for non-r. Nevertheless, in all three cases of defeaters we have pairs of competitors squaring off and the strength of their reliable indicators determine the epistemic status of the belief in question.

Turning to another example widely discussed within recent epistemology, the airport case can be expanded to provide illustrations of both kinds of defeaters.^11^ A couple about to embark on a flight to New York wonders whether the flight has a layover in Chicago. Various epistemic claims and challenges arise from different sources. That the itinerary, on which the fellow passenger bases his belief that the flight has a layover, actually is outdated, or contains a misprint, undermines that prima facie warrant irrespective of whether anyone has access to these defeaters according to the present proposal. Similarly, if the electronic billboard in the departure hall shows that the flight is direct, as an example of a stronger reliable indicator of non-p, then the alleged positive epistemic status of the fellow passenger is overridden, irrespective of whether the participants in the conversation have access to that defeater. The information on the electric billboard constitutes an overriding defeater in virtue of being a reliable indicator, irrespective of any internalist demand on access to that information. (These cases can be spelled out in different ways, see below in section 4.)

Due to the gradual nature of warrant, however, and following the considerations given above, that there exists a reliable indicator for non-p does not entail that the ground the subject bases her belief that p on is not a reliable indicator. Overriding defeat of p thus does not entail lack of *any* warrant for p even above the threshold for warrant, only that the stronger warrant rules the day when the *total* warrant is assessed. (See also section 4 below.)

These examples illustrate how, in assessing the relevant totality and ceteris paribus, itineraries without misprints provide stronger warrant than itineraries containing misprints. The addition of a no defeater-condition to warrant can thus be given a purely externalist ground.^12^ Hereby, we arrive at a unified externalist model of warrant and defeat enjoying the fruits of internal coherence, simplicity and theoretical integrity. We see that even though undermining defeaters work in more complicated ways than overriding ones, the conditions determining whether p or non-p, g or non-g or r is warranted or defeated are, nonetheless, the same.

The present proposal is much influenced by William Alston’s writings on his reliable indicator version of reliabilism especially (Alston 1989a), partly because focusing on the reliability of the *ground* rather than the *process* of belief formation more aptly fits an externalist account of defeat by providing a fruitful externalist substitute for evidence.^13^ However, my proposal is more externalist than Alston’s in two respects. (I) Unlike Alston’s hybrid account of justification, access to the ground does not constitute a necessary condition on warrant (or defeat) on my account. (II) As pointed out above, Alston also poses internalist constraints on defeat,^14^ while I do not. Note thus that the term “indicator” is not intended to convey that the subject herself can employ the ground as an indicator of truth.^15^ The choice of that term should instead be understood as conveying the *fallibility* of warrant by indicating rather than entailing the truth of the belief.

In line with the focus on defeat in this paper, I am also much in sympathy with adding a contextualist element in the form of competitors as Marshall Swain does in (Swain 1981). I suggest that his notion of relevant competitor can fruitfully be spelled out with the three kinds of challenges and defeat I outline here. The epistemic status of a belief is dependent on what challenges can be raised in the context where the belief is formed and held. The proposed account thus contains a contextualist element. More precisely, my proposal primarily bears affinity to the so called *inferentialist* version of contextualism, associated with Michael Williams, rather than the more common attributor versions of contextualism.^16^

To further spell out my proposal, it is useful to compare it with the notion of a *factual*, also called propositional, defeater employed by some epistemologists. “A factual defeater is a true proposition such that if it were added to S’s belief system, then S would no longer be justified in believing that p”.^17^ Factual defeaters thus defeat by being true. An adapted example would be the true proposition that “there are some other birds, indistinguishable to S from swans, where S made her sightings”, which make beliefs S form about swans unwarranted. Call that proposition O. In my terminology this example constitutes a candidate for an undermining defeater, more precisely of r. O undermines S’s sightings of presumed swans so that these sightings do not warrant the beliefs S forms about swans, i.e. g does not stand in relation r to p. (Alternatively, this type of defeat could be cases of non-g, if it was the sighting of a bird of the non-swan sort that S based her belief on, but I infer from the context that this kind of defeat is not what these epistemologists have in mind.) However, in order to constitute an undermining defeater of r it is neither necessary nor sufficient that the proposition is true (nor for that matter that the proposition is added to S’s belief system).

First, the truth of O is not a necessary condition since the presence of reliable indicators of these other birds constitutes warrant for r not holding, irrespective of whether O is true. Reliable indicators may exist for falsehoods as well. Second, it is not a sufficient condition since the mere addition of O to the belief system of the subject does not defeat the warrant, even if O is true. Just as the mere addition of a true belief without any positive epistemic status does not make a subject warranted in some belief, defeat only occurs when the belief is based on warrant in form of a reliable indicator. Once again, if accounting for defeat solely consisted in upholding internal rational coherence, the mere presence of a belief would call for revision if its addition would yield incoherence in the belief system of the subject. But reasons for rejecting that view has already been offered and further reasons will be given in the next section.

Finally, as long as we confine ourselves to the epistemic standing of warrant, the same argument can be run for a factual overriding defeater, although of course the epistemic standing of knowledge will never obtain when the belief is false.

Against this outline of an externalist account of defeaters, two objections are here going to be considered in the next and final section.

## Two Objections

Firstly, by posing these externalist conditions on defeat, the account becomes too open. More specifically, there will always be defeaters lurking around in the periphery ensuring that no one will ever be warranted in believing anything (even if we restrict our assessment to a particular point in time, as we should), which is a counterintuitive consequence. Moreover, should not subjects realize their unfortunate predicament and adjust their prima facie assessments accordingly?^18^ In other words, we should all regard ourselves as lacking warrant for any of our beliefs, which is a highly counterintuitive and undesirable result. In response, further flesh to the bone is undoubtedly needed, but in the meantime two points can be made.

As my main reply, I would like to emphasize that defeaters do not come cheap, but has to fulfill the same conditions of adequacy as those posed on warrant, more precisely those suggested above. It is not sufficient to defeat the epistemic status of a belief that p on the mere suspicion of a challenge lurking, just as the mere hope of the existence of warrant is not sufficient for a subject being warranted in a belief. Only the presence of (sufficiently strong) reliable indicators, in any of the aforementioned three forms, warrants or defeats the epistemic status of our beliefs. Neither warrant nor defeat comes for free. More precisely in order to defeat, the warrant for the defeater must be at least as as strong or stronger than the warrant for its competitor, i.e. the targeted belief.^19^ In order for the positive epistemic status of the targeted belief to be defeated, it must therefore be the case that there are reliable indicators present, of any of the three kinds of defeat, at least as strong or stronger than the reliable indicators on which the belief was based. For the global catastrophic scenario to occur, this must then be the outcome for every one of our beliefs! Pointing to the mere possibility of defeat skulking in the periphery leaves us far off from having established this radical conclusion.

The view on defeaters defended here with respect to epistemic strength is not without opponents. Michael Bergmann asks us to consider a case where you become convinced that you are the victim of a skeptical scenario

what happens to the justificational status of your belief that you have hands once you become convinced that you are a brain-in a-vat? Is that hand belief defeated? Or is your belief that you are a brain-in-a-vat defeated? As for that last question, it’s clear that your belief that you are a brain-in-a-vat doesn’t get defeated because we are stipulating that it never had any justification to begin with. So it can’t *lose* any justification. What about the belief that you have hands? It was justified. Does it lose its justification? I think the answer is yes.^20^

I, on the contrary, think the answer is no. Simply put, only beliefs that can lose their own warrant can make other beliefs do the same. A belief without any positive epistemic status is an idle, impotent player in the epistemic game of warrant and defeat. It constitutes a *naked* or *empty* challenge.^21^ Moreover, since the belief that you have hands possessed some previous positive epistemic strength, it wins the day over a belief with the initial epistemic strength of zero.

Bergmann offers a reply of sorts to a similar reply he calls *The Arbitrariness Objection*. He argues that we should treat warrant and defeat differently, the main reason being that “in the case of defeat, there is nothing relevantly like the transmission of some degree of epistemic status ([...] by an ideal inference). It’s not as if the belief which is a defeater has a degree of *unjustifiedness* which gets transmitted by an ideal inference to the defeated belief. Nor does the belief which is a defeater have a degree of justification which gets transmitted by an ideal inference. In cases of defeat, justification gets lost not transmitted.”^22^

Yes, of course the *targeted belief* looses its warrant in case of defeat. But it is precisely in virtue of the epistemic strength of the defeater, i.e. the epistemic strength of the competitors/challenges to p, j or r, that the positive epistemic status of the targeted belief is lost rather than preserved. No “degree of unjustifiedness” is transmitted, but the defeater does indeed possess an epistemic strength in virtue of which the epistemic status of the targeted belief is defeated. If we spell out the second option Bergmann offers in this way, then it does not appear as counterintuitive as he makes it seem like. The outcome may indeed be that the subject instead becomes warranted in believing that non-p. This happens for overriding defeaters when there is a considerable difference in epistemic strength in favor of non-p over p. In case their epistemic strength is close, however, the subject is not warranted in having any of these beliefs, but rather to suspend her original belief that p.^23^

Notice as well that the question *why* the subject should take the defeater to defeat the targeted belief, rather than, say, the other way around, does not receive a complete answer on Bergmann’s account.^24^ What if you happen to possess some warrant for the defeating belief? No complete explanation is thus offered why that specific instance of defeat occurs rather than some other, hence the outcome is precisely arbitrary. At best, Bergmann seems to paint a scenario of incoherence already dealt with in section 1.

In addition, even if it is granted that warrant will become harder to come by, which I regard as an open question, this supposedly counterintuitive consequence will primarily affect our *access* to the warrant and thus our *ascription* of epistemic states. Our own epistemic status may become more difficult to access and assess, which leads us to the second objection.

Thus as the second objection, it could be argued that an externalist account fails to take into account, not only *intra*subjective epistemic assessments as in the first objection, but also *inter*subjective assessments of fellows in the epistemic practice of forming beliefs and making epistemic claims, providing warrant for them as well as defeating those of others etc. Sometimes these participants are also awarded deontological assessments like blame and praise by their peers. It seems unavoidable that these assessments are based on accessible warrant only.

I agree that a full account of warrant and defeat should account for those assessments of epistemic status. Luckily, this can be accomplished within the present account by (finally!) explicitly introducing the notion of a *challenge*. Whereas defeaters thus possess externalist conditions, challenges instead have internalist ditto: a challenge is a prima facie defeater based on *accessible* warrant. A corresponding distinction between overriding and undermining challenges can be drawn for them as well.

In our epistemic practice we often cannot await the *ultima facie* verdict on epistemic claims we encounter, we have to cope with whatever warrant is accessible to us at the time of the assessment. Any such assessment is, however, precisely prima facie, open to future correction by new relevant warrant all the way to the final *ultima facie* stage, which is often never actually reached. Whilst any ascription of epistemic status is accordingly based on prima facie warrant, the *ultima facie* epistemic status is determined by the aforementioned externalist conditions, not necessarily accessed by anyone. For instance, in several of the examples from the former section, like the airport case, the purported epistemic status, being challenged in various ways, precisely goes through several stages of prima facie assessments before arriving at the *ultima facie* stage.

Although an internalist aspect is thus introduced into the present account with the notion of a challenge, no rival conditions for assessing epistemic status are thereby introduced and therefore no internal tension arises. The conditions are the same; the only difference concerns to what extent the warrant relevant for the assessment is available to the fellows at the time of assessment. The internalist prima facie conditions firmly rest on the externalist *ultima facie* conditions. The prima facie assessments are therefore always sensitive to new warrant, in benign scenarios moving these assessments further towards the *ultima facie* goal, which also explains why fellows of the epistemic practice are willing to revise their earlier assessments in light of new warrant. Often, as in the case of the aforementioned flight itinerary, the fellows may successively gain fuller access to the epistemically relevant features of the warrant they already base their beliefs on, like the itinerary being outdated or containing misprints. In less fortunate scenarios, however, the *prima* and *ultima facie* scenarios may come further apart and phenomena like misleading defeaters occur.

The verdict that the clairvoyants Samantha and Maud are *ultima facie* warranted in their beliefs despite facing their respective challenges, can then be complemented by the prima facie characterization of their beliefs as unwarranted since they cannot meet the challenges they are faced with. Failure to do so is indeed an *indication* of defeat. However, if and when they realize that these challenges ultimately fail in their cases, so that they come to understand that they precisely faced *misleading* defeaters all along, then they could revise the prima facie characterization and rightly classify themselves as having been warranted in their clairvoyant beliefs, not just becoming so at that moment, as a result of this realization, but ever since their reliable clairvoyant visions began.

To further boost this admittedly controversial verdict, compare by contrast with a scenario where *new* epistemically relevant factors emerge in time in either direction, say in the form of further cases of confirmation within an unfolding track record of their clairvoyant powers. In cases of that kind, their *ultima facie* epistemic status would indeed vary with these factors, but thus *not* depending on their accessibility. My solution can thus nicely distinguish misleading defeaters from so called “defeater defeaters”, i.e. subsequent defeat of a defeater,^25^ which misleading defeaters wrongly seem to be treated as, according to analyses where they are genuine until their misleading character is accessed and revealed.

In the case of Samantha, however, I would like to add a qualification, in line with considerations given above and in an earlier work of mine.^26^ If the warrant for the overriding challenges to p, i.e. for non-p, is stronger than the warrant her reliable clairvoyant powers yield for p, then she would not be warranted in continuing to believe that p, despite p being true. Defeat would thus occur. As far as I can tell from BonJour’s paper, the case is underdescribed in this crucial respect, so we need to fill in the scenario by adding the strength of the warrant for p and non-p respectively, for instance just how reliable Samantha’s clairvoyant powers are. Once again, neither warrant nor defeat comes for free. The bare presence of “apparent cogent evidence” for non-p is thus not enough to defeat the epistemic status of p.^27^ However, if the warrant for non-p wins the day, then that outcome should *not* be characterized as a misleading defeater, but as a genuine one. Unlike the case of misleading defeaters, no split here occurs between the accessible and non-accessible epistemic factors. The warrant for non-p is *ultima* facie stronger than the warrant for p. Instead, such cases constitute negative analogues to cases of genuinely warranted beliefs that, nonetheless, are false. Cases of the latter kind can occur within a fallibilist framework and we should not be surprised that the same phenomenon can occur in cases of defeat.

Despite ultimately biting the bullet with the positive verdict on their *ultima facie* epistemic status (with the qualification I just added), the introduction of the notion of a challenge thus allows me to at least partly accommodate the intuition that the clairvoyants are unwarranted in their beliefs. For those who regard the primary role of defeaters as managing epistemic rationality and thus not find my externalist account useful in this regard, my reply is that my account provides the groundwork for a model of epistemic rationality by supplying the conditions under which the epistemic standing of the subjects’ beliefs ultimately are awarded and thus *what* features to look for to the best of one’s abilities when intra- or interpersonal conflicts arise in the belief systems of epistemic agents.

These verdicts and considerations seem reasonable to me, unsurprisingly, but are probably not in accord with everyone’s intuitions. However, since no model can incorporate every intuition, at some point, our verdicts must be theory driven. Thus in the face of overriding challenges against the present account in the form of such conflicting intuitions, the present account relies on the theoretical virtues of unity, simplicity and integration.^28^

Having thus shown how these internalist intuitions can be at least partly reconciled within my account, I want, nonetheless, to draw a limit to its accommodation. I am disinclined to introduce deontological evaluations on the states and moves of the fellows in the epistemic practice since I take Alston’s classical criticism of deontological conceptions of warrant to heart.^29^ Granted, the present account of warrant, challenge and defeat does not prohibit characterizing certain states as blameless or blameworthy, but I regard such characterizations as uninformative in that, say, blamelessness does not add any epistemically relevant property to warrant once the aforementioned conditions of warrant are in place, whereas if these conditions are omitted, then it is difficult to distinguish *epistemic* blamelessness from other forms of blamelessness; and, second, that deontological evaluations are misleading by precisely suggesting a voluntary control over our beliefs that we lack – just as Alston convincingly argued.^30^

By drawing attention to the limits of incorporating deontology into the present proposal, my investigation comes to an end. While we have not obtained complete clarity concerning the nature of defeaters, we have reached a deeper understanding of the epistemic properties that determine defeat. Thus we have made progress towards developing a unified externalist account of both warrant and defeat.

## Footnotes

1 For lack of a better term, in this paper *warrant* is the *genus* of whatever epistemic property fulfills the third condition beyond true belief in the classical tripartite definition of knowledge and is neutral with respect to any externalist or internalist constraints. Note that warrant in my terminology does not handle Gettier cases. The Gettier problem is not addressed in this paper. (A helpful suggestion by an anonymous referee clarified my terminology here.) *Justification* is, then to take an example, a *species* of warrant to which internalist constraints are attached.

2 Greco notes this internalist tendency and gives several examples of mixed positions in Greco 2010 sec. 10.1. One of them is (Bergmann 1997, 2006), whom I shall discuss in more detail in sec. 4 below. More precisely, in the former paper Bergmann exempts the no defeater condition (NDC) from the externalism-internalism debate. His preferred form of externalism “is the view that there is no internal condition other than NDC that is included among those severally necessary and jointly sufficient for warrant.” (p. 407). I have the ambition here to instead include NDC on *externalist grounds*. I thereby take issue with BonJour’s view that NDC cannot be motivated on externalist grounds (BonJour & Sosa 2003, Part 1, Ch. 2, sec. 2.3) while agreeing with him that for externalism to make internalist concessions regarding NDC “amount to an untenable halfway house” (ibid. p. 32). (Thanks to an anonymous referee for urging clarification here.)

3 Somewhat in the spirit of Goldman’s recent treatment of evidence in Goldman 2015. See note no. 13 below. But of course I hope other readers also will benefit from reading the paper!

4 See Pollock and Cruz 1999, 196 and Casullo 2003, 44–5. (Pollock and Cruz employs the terms “rebutting” for overriding and “undercutting” for undermining defeaters.)

5 For reasons given below, the term “challenge” is used here rather than “defeater”. In the terminology of this paper, a *challenge* differs from a defeater by constituting a prima facie epistemic state. See sec. 4 and Janvid 2013﻿. As is noted in that work the problem of misleading defeaters is most often phrased in terms of knowledge, but can anyway instructively be employed for my purpose. Note that the defeater is misleading in the sense of leading the subject to believe that p is false when in fact p is true because the subject lacks access to all the epistemically relevant aspects. The defeater is not misleading in the sense of the accessible aspects somehow being misunderstood by the subject. It is the lack of access to other epistemically relevant aspects that mislead the subject. See below in the main text and the proviso added in sec. 4. (I am grateful to an anonymous referee for urging clarification here.)

6 Not that diverging externalist verdicts on these thought experiments necessarily rest on this tendency. For three such well argued treatments that focus on other problematic features of BonJour’s thought experiment, see Greco 2010 sec. 9.2, Henderson and Horgan 2011, Ch. 3, sec. 11 and Lyons 2009, Ch. 5.

7 BonJour 1980, 59–62. The second case figuring Casper, who has repeatedly found his past clairvoyant predictions defeated but this time gets it right, is harder to classify as misleading, as well as whether the alleged defeater is an overriding or undermining one. Without using this distinction, BonJour seems to treat the case of Casper as an undermining one (more precisely of g according to the distinction drawn above). This classification is in accordance with my analysis: the bad track record undermines the warrant rather than directly the confirmed prediction. Next, is the warrant for Casper’s prediction really defeated by the track record? It seems not since the bad track record is a misleading one, the predictions were defeated, not by his clairvoyant powers being unreliable up to the actual prediction, but because of various external circumstances. The fourth case involving Norman does not contain any defeaters. He simply lacks any positive reason to trust his clairvoyant beliefs. The terminology employed here as well as the verdicts are further substantiated in sec. 4 below.

8 Burge 2013, 26–7.

9 Bonjour considers and concedes this point in BonJour 1980, 66–7, but does not think that it carries much weight unless one could establish a stronger defense on common sense or show that alternative approaches fail in this regard. My externalist response is (i) that common sense is not sacrosanct per se, but rather that it is in line with the externalist condition on warrant in this case and (ii) that the main rival internalism precisely fails in this regard just as Bonjour concedes. The warrant track records provides are, of course, ultimately circular in this regard. It has been pointed out to me that the analogy between clairvoyance and perception assumes that the clairvoyant beliefs have a ground that they are based on just as perception has. I confess that this is the only way to make sense of clairvoyance for me; but, moreover and more importantly, unless some such mechanism, as well as an input to that mechanism, exists to generate the beliefs about the whereabouts of the president, the cases do not constitute a counterexample to externalism in general and reliabilism in particular. Note no. 12 in (ibid.) supports this interpretation. Greco allows the clairvoyant subjects to be warranted, and thus the defeaters to be misleading, as long as they have very different cognitive characters than we do in Greco 2010 sec. 9.2. It is very hard to accept the premises of the thought experiments unless they do differ from us to a large extent.

10 The ground thus enjoys higher adequacy in Alston’s terminology from Alston 1989a. The present proposal is arguably more in spirit with Alston’s view than his own internalist proposal regarding the “no defeater”-condition. See below. Truth conduciveness and probability are of course difficult and controversial philosophical concepts. I have regrettably at present nothing to add over above Alston’s attempt to give an account of objective probability within epistemology in Alston 2005. See also note no. 13 below.

11 See Janvid 2008 and 2013.

12 While in general sympathetic to a unified externalist approach, Greco in Greco 2010, Ch. 10 rejects the view that “defeat relations supervene on probability relations among the propositional contents of the beliefs involved” more formally (p)(q)[{P(p/q) < .5}&~∃(r){P(p/q&r) > .5}⇒qDp] (161) since this view leads to untenable skepticism (164–6). (D stands for the defeat relation.) In support he offers three examples where the antecedent is fulfilled but where p, nevertheless, is not defeated. Three subjects, on the one hand, endorse common sense beliefs, but also, on the other, contemplate radical philosophical views that they are unable to refute like that time, physical objects or moral responsibility are not real. Nonetheless, the subjects have knowledge of these commonsense propositions. In response, it could be argued that, first, if these subjects really fulfilled the antecedent of the principle, then it would not be so unreasonable to hold that their commonsense beliefs were defeated. Unlike these three subjects however, most subjects reflecting on these intricate philosophical arguments and finding themselves unable to refute them, nonetheless do not believe in their conclusion to the extent that the antecedent is true. Second, *if* the philosophical arguments were sound, and thus their conclusions true, then the three subjects, like the rest of us, would *not* know these commonsense propositions. We believe and hope that they are not and that a refutation will come along any century now!

13 In this regard, Goldman in Goldman 2015 expresses his disaffection with his own original suggestion concerning defeat in terms of available alternative reliable processes, describing that approach as “strained” (note 15). He now instead sees interesting parallels between evidence and reliable indicators and thinks doing so will better handle, for instance, BonJour’s reliable clairvoyants. I myself find this new approach promising and his comparison between the two notions elucidating and broadly in line with my suggestions in this paper. However, Goldman (i) does not go on to develop defeat itself in terms of reliable indicators (ii) does not introduce competitors into his presentation of reliable indicators as I do (iii) seems to regard the reliable indicators as internal mental states of the subject (but that may be to suade evidentialists), and finally (iv) also seems to treat the notorious clairvoyants as unjustified(?)

14 ”[T]he fate of prima facie justification is determined by what is in the subject’s perspective on the world, rather than by the way the world is” (Alston 1989a, 239). The internalist constraint is, however, of a weaker kind than accessibility (perhaps more in line with mentalism?).

15 As in the case of Chase 2004. (Thanks to an anonymous referee for urging clarification here).

16 See, for instance, Williams 2001 and Janvid 2013 where some friendly amendments are suggested. Attributor contextualism is criticized in the same work.

17 Lackey 2008, 45–6 note 21. See also Bergmann 2006, 153–5. Both contrast factual defeaters with *mental state defeaters* that specifically defeat justification on their views. It should be clear from this section, that constituting a mental state is neither necessary nor sufficient for defeating warrant on my account. For further criticism of the notion of factual defeaters, see Grundmann 2011, 157. Concerning mental state defeaters, he objects to their necessity, i.e. that “there is only one source of defeaters, namely an internal lack of rationality” (161), but nevertheless accepts their sufficiency. Grundmann himself subscribes to a version of proper function-externalism for defeaters, a general framework I myself find unpromising, one reason being that I am suspicious towards the epistemological relevance of etiology.

18 Klemens Kappel raised these concerns in Bled.

19 See Janvid 2013 and sec. 3 above about competitors.

20 Bergmann 2006, 165, italics in original.

21 See Janvid 2008 and 2013 and Grundmann 2011, 164–5. The notions naked or empty challenge derive from Williams who now calls them *free* challenges (Williams 2011, 50–1.) Here is thus an example of a point in which I am in sympathy with inferentialist contextualism.

22 Bergmann 2006, 167 italics in original.

23 See Janvid 2013.

24 Unless we uncharitably take to heart what Bergmann states in the first quote that only beliefs that possess justification can be defeated whereas beliefs lacking justification cannot be, which suggests a radical epistemic recipe for avoiding defeat.

25 Grundmann 2011, 161–2.

26 In Janvid 2013.

27 BonJour 1980, 59. By contrast, the undermining challenges directed against g for Maud seem more straightforward: the challenge is prima facie warranted, but due to factors underdescribed in the thought experiment, the general suspicions against clairvoyance does not hold for Maud and (*ultima facie*) defeat does not occur.

28 What about misleading positive warrant for p? Do they make the subject *ultima facie* warranted in a belief that p? Admittedly, a reliable indicator can, nonetheless, make us genuinely warranted in a false belief, but just as in the case of misleading defeaters investigated in the main text, an indicator which appears to be reliable, but in fact is not, does not make us warranted in a belief (irrespective of whether the belief in question is true or false).

29 Alston 1989b and, more recently Alston 2005, Ch. 4.

30 See Janvid 2009 for criticism of Dretske’s attempt to explicate warrant in terms of responsibility and further discussion of blamelessness as an epistemic state.
